# Spectral CT-derived peripheral iodine concentration in acute pulmonary embolism: association with subsegmental emboli and pulmonary infarction

**DOI:** 10.1186/s41747-026-00784-1

**Published:** 2026-09-08

**Authors:** Agreen Horr, Schekeb Aludin, Lars-Patrick Schmill, Karim Mostafa, Isabella Luisa Walther, Domagoj Schunk, Hannes Peckolt, Olav Jansen, Patrick Langguth

**Affiliations:** 1 Department of Radiology and Neuroradiology, University Hospital Schleswig-Holstein, Campus Kiel, Kiel, Germany; 2 Interdisciplinary Emergency Department, University Hospital Schleswig-Holstein, Campus Kiel, Kiel, Germany; 3 Clinician Scientist Program, Faculty of Medicine, University of Kiel, Kiel, Germany

**Keywords:** Angiography, Iodine, Pulmonary embolism, Pulmonary infarction, Tomography (x-ray computed)

## Abstract

**Objective:**

Pulmonary infarction (PI) is a recognised complication of acute pulmonary embolism (PE). While computed tomography pulmonary angiography (CTPA) provides high-resolution morphological imaging, it does not permit direct functional assessment of regional lung perfusion. Spectral CT pulmonary angiography (SCTPA) enables quantitative perfusion analysis using iodine maps. However, the relationship between peripheral perfusion impairment, subsegmental emboli (SSE), and PI remains incompletely understood. This study investigated their association using SCTPA.

**Materials and methods:**

This retrospective single-centre study included 62 patients: 31 with SCTPA-confirmed acute PE and at least one PI, and 31 controls without PE or PI. Peripheral iodine concentration (pIC) was quantified in standardised subpleural regions of interest in each lung segment. Embolic burden was assessed using the Mastora score. SSE were identified on SCTPA and analysed on a segmental basis.

**Results:**

A total of 440 embolic occlusions were identified. In this selected cohort, PI occurred exclusively in segments affected by SSE and was not observed in segments without SSE. In mixed-effects modelling, SSE were independently associated with reduced pIC (β = -0.706, *p* < 0.001), while segmental emboli were also independently associated with lower pIC (β = -0.137, *p* < 0.001); lobar and central emboli showed no significant independent associations. The Mastora score showed a moderate correlation with the number of manifest PI (ρ = 0.566, *p* = 0.011).

**Conclusion:**

In this cohort, SSE were closely associated with PI and reduced peripheral iodine concentration. Quantitative pIC reflects regional hypoperfusion and provides functional information on lung perfusion in acute PE.

**Key Points:**

**Question:** Can spectral CT-derived peripheral iodine concentration characterise regional perfusion impairment associated with subsegmental emboli and pulmonary infarction in acute pulmonary embolism?

**Findings:** In this selected cohort, pulmonary infarction occurred only in segments with subsegmental emboli, which showed reduced peripheral iodine concentration (pIC) on spectral CT iodine maps.

**Relevance statement:** Spectral CT-derived peripheral iodine concentration may provide functional information on regional hypoperfusion in acute pulmonary embolism and improve understanding of infarction-related perfusion impairment.

**Graphical Abstract:**

**Figure:**
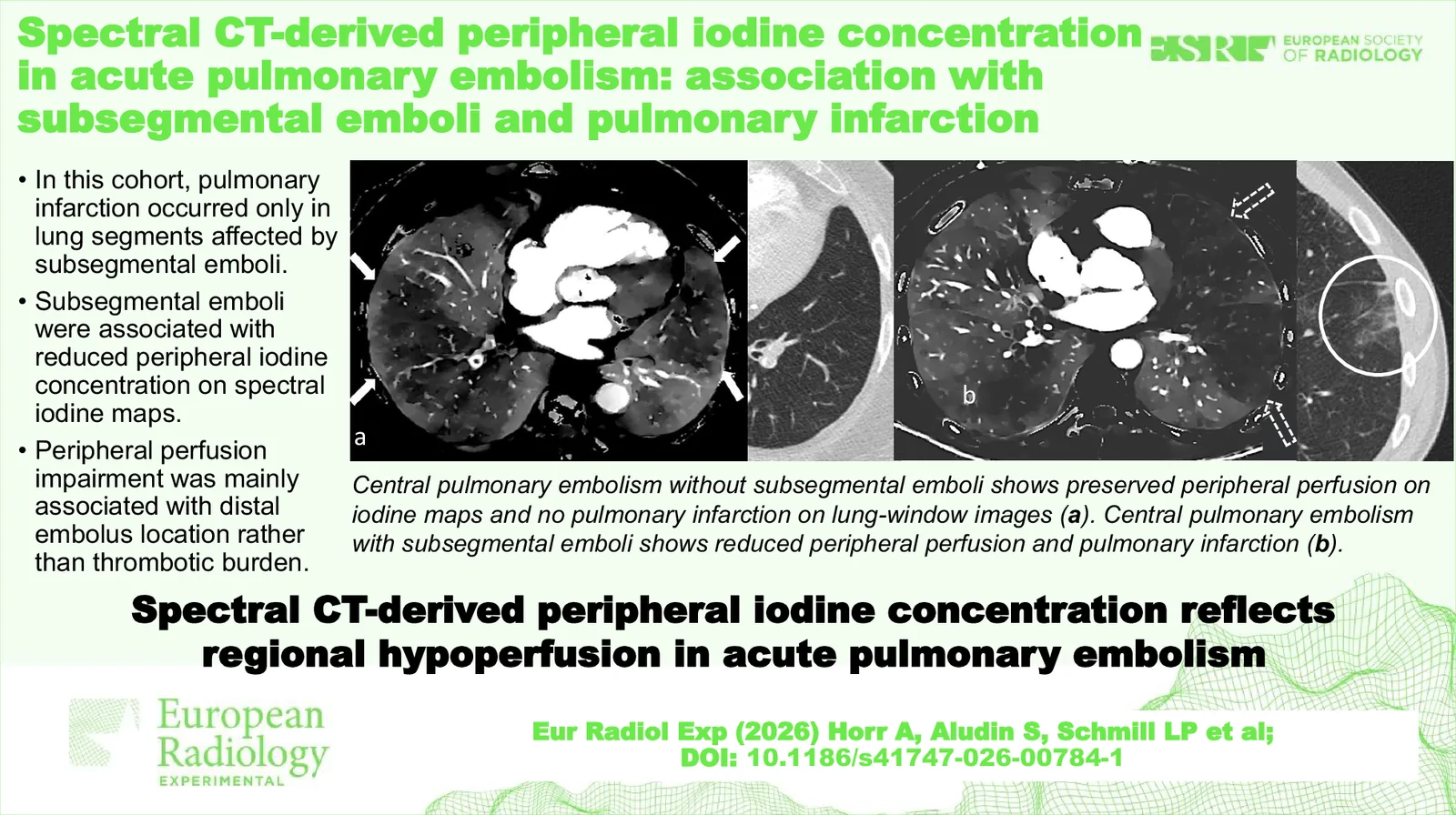

## Background

Pulmonary embolism (PE) is one of the most common acute cardiovascular diseases [1]. It usually results from obstruction of a pulmonary artery by thromboembolic material originating from the deep veins of the lower limbs or pelvis [2], and it frequently necessitates inpatient treatment and, in severe cases, intensive care treatment [3]. A clinically relevant complication of PE is pulmonary infarction (PI) [4–6], defined as haemorrhagic necrosis of the lung parenchyma due to prolonged ischaemia [4, 7]. Despite the lung’s dual blood supply *via* the pulmonary and bronchial arterial systems [8, 9], the reported prevalence of PI in acute PE varies widely, ranging from 9% to 68%, depending on the study population and imaging modality [5, 6, 10–15]. Radiologically, PI typically presents as a subpleural, wedge-shaped consolidation, but differentiation from atelectasis or inflammatory infiltrates may be challenging [4, 7].

The development of PI is largely determined by the capacity of the bronchial arterial circulation to compensate for impaired pulmonary perfusion [8, 9]. Due to its systemic origin and its mean arterial pressure being approximately six times higher [16], the bronchial circulation can often preserve adequate parenchymal perfusion in cases of central PE through extensive bronchopulmonary anastomoses [17, 18]. In contrast, in peripheral lung regions, the collateral vascular network is less developed [17], and its compensatory capacity is markedly limited in segmental and, in particular, subsegmental arterial occlusions. In these settings, increased bronchial blood flow may result in alveolar haemorrhage, which can progress to infarction if resorption is insufficient [4, 7].

Although computed tomography pulmonary angiography (CTPA) is the gold standard for diagnosing PE, it primarily enables the detection of intraluminal thromboembolic material and does not provide direct information on regional lung perfusion [19]. Spectral CT pulmonary angiography (SCTPA) extends conventional CTPA by acquiring energy-resolved datasets that enable improved material characterisation [20–24]. Iodine maps derived from these datasets visualise the regional distribution of contrast material within the lung parenchyma and allow direct assessment of parenchymal perfusion. As a result, SCTPA provides both morphological and functional information in a single examination [20–24].

Perfusion distal to an occluded pulmonary artery is of particular interest, as these regions are expected to exhibit critical hypoperfusion. However, it is unclear to what extent quantitative measurements of peripheral iodine concentration (pIC) reflect regional perfusion alterations and their association with the occurrence and severity of PI.

The aim of this retrospective, single-centre study was therefore to investigate the relationship between peripheral lung perfusion and the presence of PI using SCTPA. Specifically, we assessed whether peripheral perfusion impairment is associated with subsegmental emboli (SSE) and infarction status on a segmental level, and how these findings relate to overall thrombotic burden as quantified by the Mastora score [25].

## Materials and methods

### Study design and population

This retrospective single-centre observational study was approved by the Ethics Committee of the Christian-Albrechts-University of Kiel (reference D 567/18; amendment 7 October 2024). Written informed consent in the form of a GDPR-compliant broad consent was available for all included patients.

Potential cases with PI were retrospectively identified through a keyword-based search of radiology reports in the institutional radiology information system among spectral CT pulmonary angiography (SCTPA) examinations performed between April 2021 and April 2024 in the emergency department of a German university hospital for suspected acute PE. Search terms included “pulmonary infarction”, “lung infarction”, and “infarction pneumonia”. All identified examinations were subsequently reviewed to confirm the imaging findings. This approach was intended to identify reported and morphologically confirmable cases of PI rather than to estimate the overall prevalence of PI among all SCTPA examinations performed for suspected acute PE.

Inclusion criteria were: confirmed acute PE with concomitant PI on SCTPA; availability of a complete SCTPA dataset including spectral base images; availability of a valid GDPR-compliant broad consent.

Exclusion criteria were parenchymal or anatomical limitations (pleural effusion, malignancy, atelectasis, pneumonia, pulmonary fibrosis, hiatal hernia, elevated hemidiaphragm); technical limitations (beam-hardening artefacts, motion artefact, missing or incomplete spectral base image dataset, inadequate contrast opacification); and administrative exclusion for missing documented General Data Protection Regulation (GDPR)-compliant broad consent (https://eur-lex.europa.eu/eli/reg/2016/679/oj/eng).

To provide reference values for quantitative perfusion analysis, a control group of patients without PE or PI was selected from the same pool of SCTPA examinations performed for suspected PE. Potential control cases were identified through the radiology information system by selecting examinations in which PE had been ruled out. All selected cases were subsequently reviewed to confirm the absence of PE and pulmonary infarction, and the same exclusion criteria as applied to the PI cohort were used.

### Imaging protocol and image reconstruction

All SCTPA examinations were performed using a 128-slice dual-layer spectral CT system (IQon Spectral CT or Spectral CT 7500, Philips Healthcare). Intravenous contrast administration consisted of 50 mL of iodinated contrast medium (300 mg I/mL; Bracco Imaging S.p.A.), followed by a 40-mL saline flush, both injected at a flow rate of 4 mL/s. Bolus triggering was initiated once attenuation in the main pulmonary artery reached 180 HU, with an approximate trigger delay of 5 s.

Acquisition parameters included a tube voltage of 120 kVp with automated mA modulation, collimation of 0.625 mm, a pitch of 1.7, and a rotation time of 0.27 s. Images were reconstructed with a slice thickness of 2 mm and a 512 × 512 matrix. Spectral raw data were exported as spectral base image files, from which material-specific iodine maps were generated for quantitative perfusion assessment.

### Image analysis

Two radiologists with 4 and 6 years of experience in thoracic imaging evaluated all datasets. The assessment included both morphological and quantitative parameters. The readers were not formally blinded to the presence of embolus or to the study hypothesis during image evaluation. The Mastora score [25], SSE detection, and PI classification were assessed in consensus; therefore, no separate interobserver agreement analysis was available for these morphological assessments.

#### Embolic burden

Thrombotic obstruction of the pulmonary arterial tree was quantified using the Mastora score, which assesses 31 predefined vascular segments. Each segment is rated from 0 to 5 according to the degree of luminal occlusion, resulting in a maximum possible score of 155 [25]. The score comprises 5 central arteries (main pulmonary trunk, right and left pulmonary arteries, and right and left interlobar arteries), 6 lobar arteries (right upper, middle, and lower lobes; left upper, lingular, and lower lobes), and 20 segmental arteries (10 per lung corresponding to anatomical segments). The Mastora score was assessed in consensus by the two radiologists.

#### Subsegmental emboli (SSE)

As subsegmental vessels are not included in the Mastora score, the presence of SSE was assessed separately. For each lung segment, SSE were recorded dichotomously (1 = present, 0 = absent). The presence of SSE was assessed in consensus by the two radiologists.

#### Pulmonary infarction (PI)

**Fig. 1:**
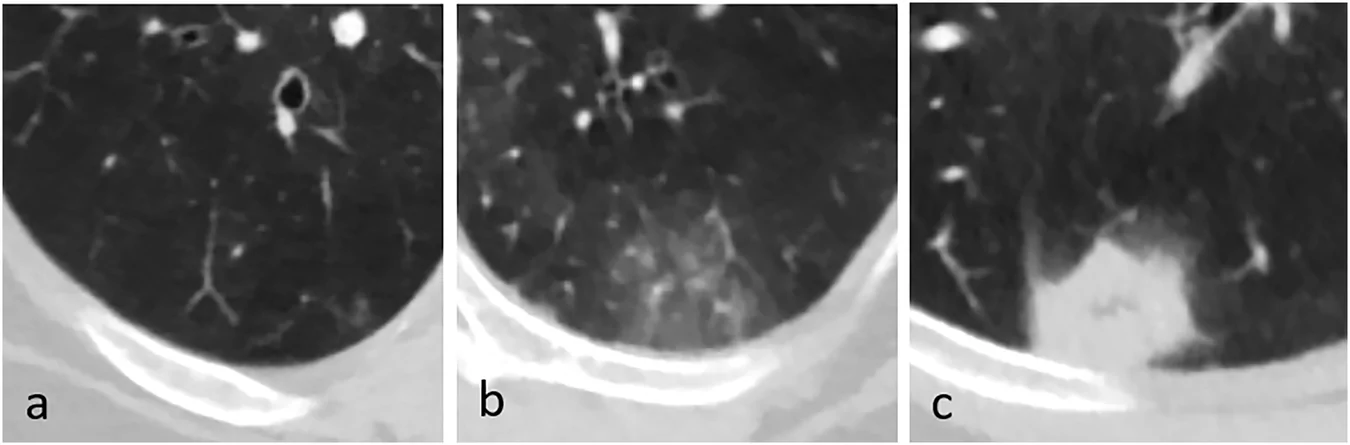
CT depiction of different stages of PI. Representative CT images displayed in lung window settings illustrate the morphological spectrum of PI. **a** Lung segment without evidence of PI. **b** Early-stage PI presenting as a subpleural wedge-shaped ground-glass opacity. **c** Manifest PI presenting as a subpleural wedge-shaped consolidation. CT, Computed tomography; PI, Pulmonary infarction

Each lung segment was classified as showing no PI, early-stage PI, or manifest PI based on morphological criteria on CT images in lung window settings (Fig. 1). The overall morphological assessment was guided by the classical descriptions by Hampton and Castleman [7] and Fleischner [26]. In the present study, manifest PI was operationally defined as a consolidated, wedge-shaped subpleural opacity with its base adjacent to the pleura. Early-stage PI was study-defined as a subpleural wedge-shaped ground-glass opacity without complete consolidation. Segments without these findings were classified as infarction-free. The classification of pulmonary infarction was assessed in consensus by the two radiologists.

#### Peripheral iodine concentration (pIC)

**Fig. 2:**
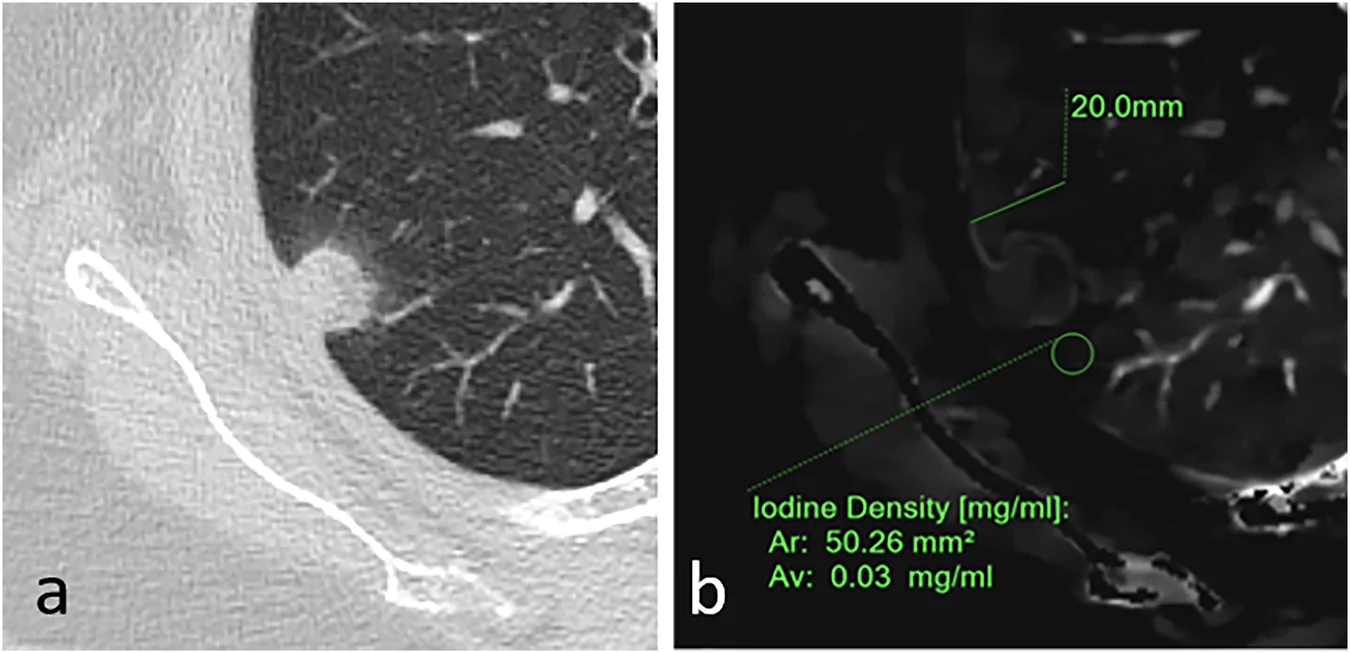
Quantification of pIC. **a** Conventional CT image displayed in lung window settings demonstrating PI, and (**b**) the corresponding spectral CT iodine map. The pIC was quantified using predefined regions of interest (ROIs; 50 ± 5 mm²) placed within a maximum distance of 20 mm from the pleural surface. ROIs were positioned within intercostal spaces to minimise artefacts. CT, Computed tomography; pIC, Peripheral iodine concentration; PI, Pulmonary infarction; ROI, Region of interest

Segmental lung perfusion was quantified on iodine maps by placing four standardised regions of interest (ROIs) per lung segment, each measuring 50 ± 5 mm². ROI placement was performed manually according to a predefined protocol on axial iodine maps. ROIs were positioned within ≤ 20 mm of the pleural surface (Fig. 2), defining a subpleural perfusion zone and ensuring consistent sampling of peripheral lung parenchyma across segments. Within this zone, ROIs were placed according to anatomical criteria and systematically distributed to obtain a representative sampling of the peripheral parenchyma, while avoiding visible vessels, bronchi, consolidation, and artefacts. For each segment, the mean pIC was calculated and normalised to the corresponding segment-specific reference value derived from the control group, accounting for physiological regional perfusion differences and reducing interindividual variability. ROI placement and pIC measurements were performed independently by the two radiologists. This standardised approach was applied consistently across all lung segments.

### Statistical analysis

**Fig. 3:**
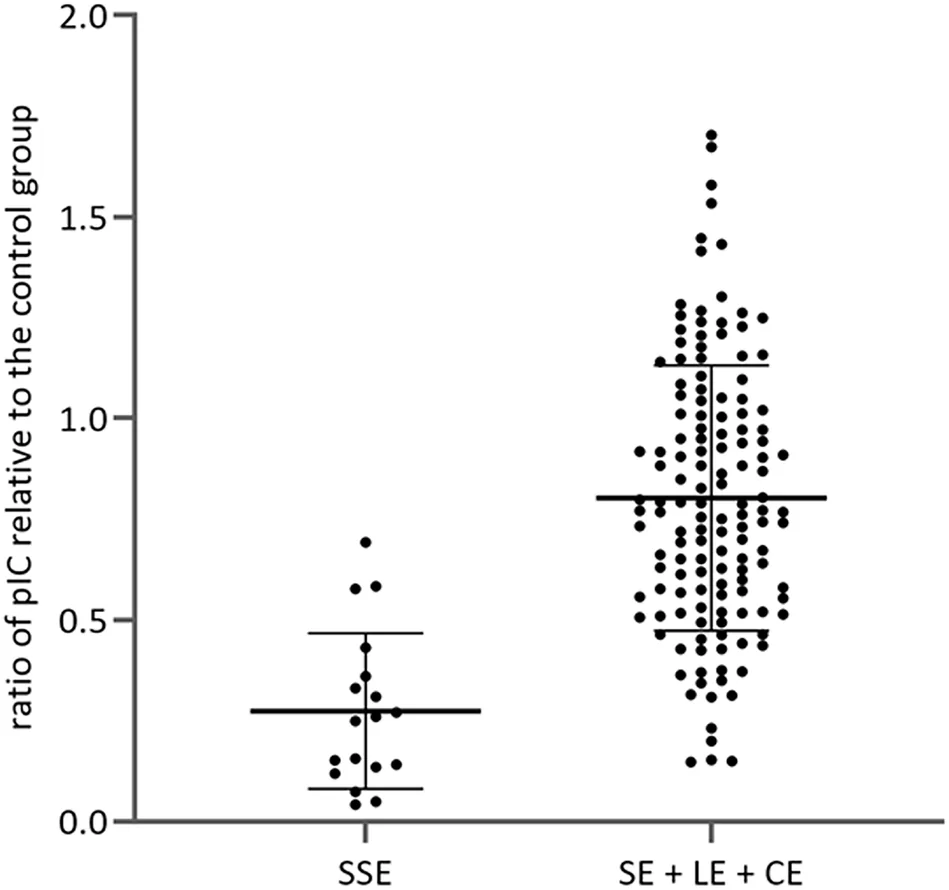
Comparison of pIC ratio between isolated SSE and more proximal emboli without subsegmental involvement. The figure shows the pIC ratio in lung segments with isolated SSE and in segments affected by emboli at more proximal levels (SE, LE, or CE) without subsegmental involvement. Statistical inference was based on a figure-specific mixed-effects model. CE, Central emboli; LE, Lobar emboli; pIC, Peripheral iodine concentration; SE, Segmental emboli; SSE, Subsegmental emboli

**Fig. 4:**
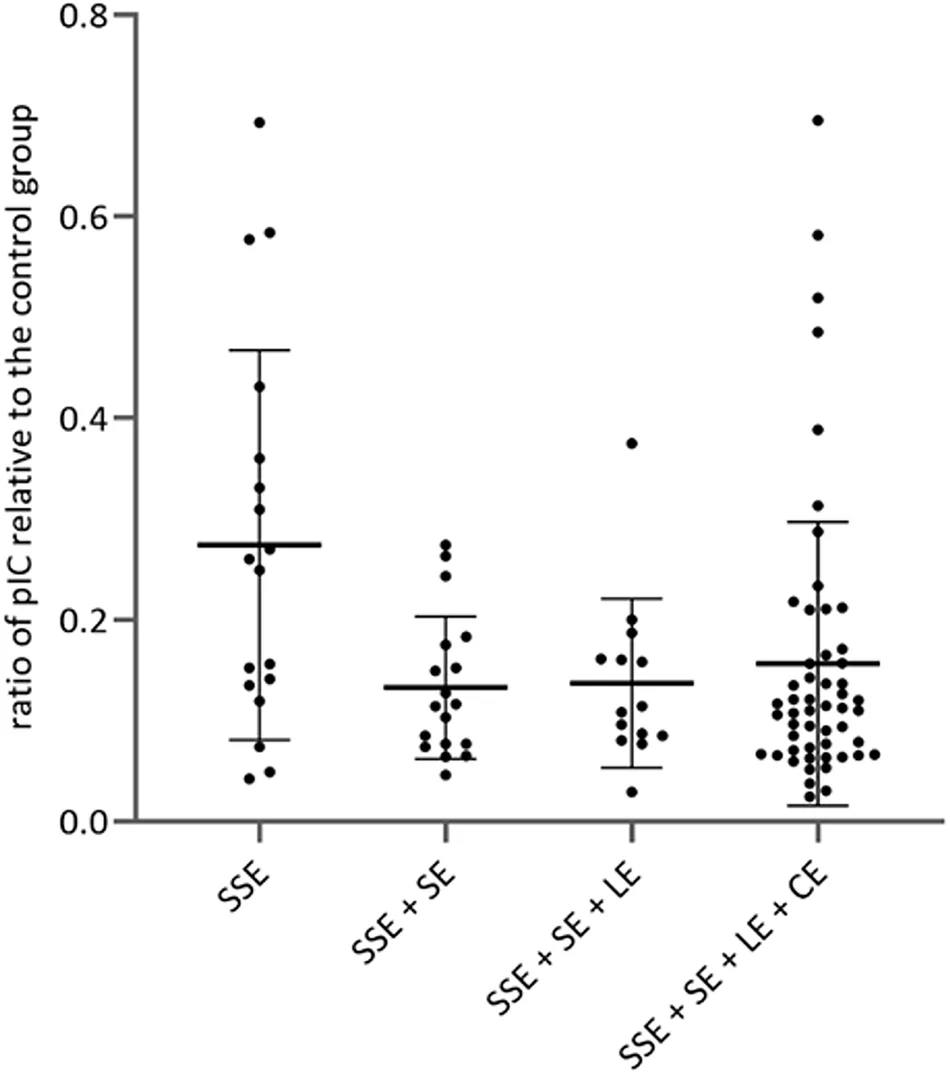
pIC ratio according to embolus pattern among SSE-positive segments. The figure illustrates the pIC ratio in lung segments with isolated SSE compared with segments showing additional proximal embolic involvement (SSE + SE; SSE + SE + LE; SSE + SE + LE + CE). Statistical inference was based on a figure-specific mixed-effects model. Pairwise *post hoc* comparisons were performed using Tukey adjustment. CE, Central emboli; LE, Lobar emboli; pIC, Peripheral iodine concentration; SE, Segmental emboli; SSE, Subsegmental emboli

**Fig. 5:**
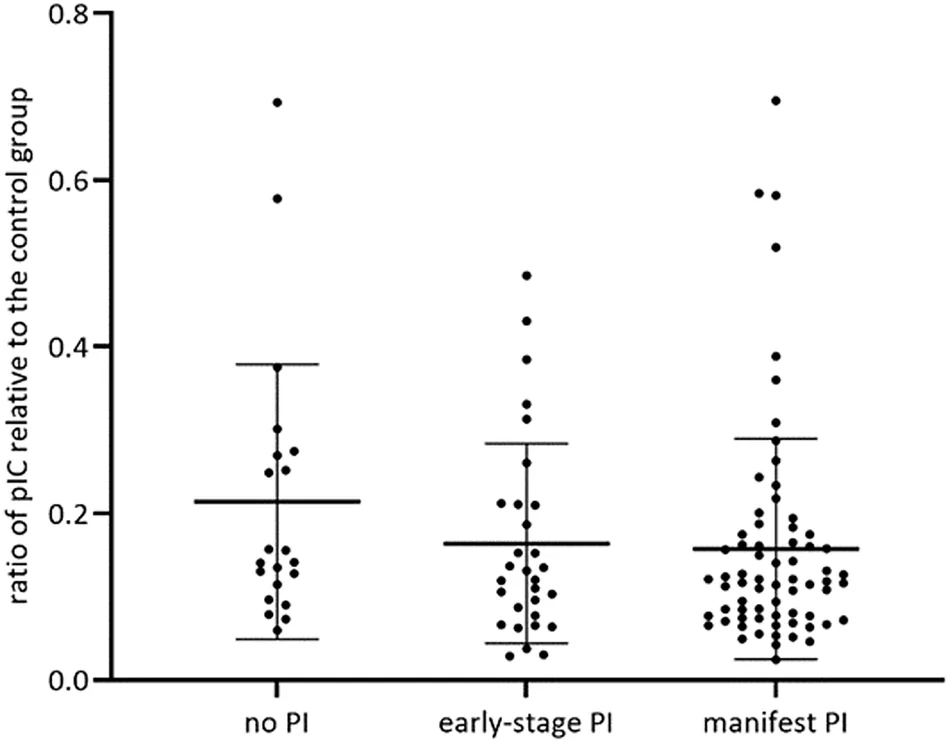
pIC ratio according to infarction status in SSE-positive segments. The figure illustrates the pIC ratio in lung segments affected by SSE, stratified by infarction status (no PI, early-stage PI, or manifest PI). Statistical inference regarding the association between infarction status and pIC ratio was based on a figure-specific mixed-effects model. pIC, Peripheral iodine concentration; PI, Pulmonary infarction; SSE, Subsegmental emboli

Statistical analyses were performed using GraphPad Prism 10 (GraphPad Software) and R (version 4.5.3; R Foundation for Statistical Computing). In the control group, segmental pIC values were analysed using two-way repeated-measures ANOVA to assess left-right differences, while cranio-caudal differences were assessed using one-way ANOVA followed by Tukey’s *post hoc* test. Inter-reader agreement for pIC measurements was assessed using the intraclass correlation coefficient. For segment-based analyses in the PI group, linear mixed-effects models were used as the primary inferential approach to account for intra-patient clustering, with patient ID included as a random intercept. In a global model, the presence of SSE, segmental emboli (SE), lobar emboli (LE), and central emboli (CE) was included as fixed effects. In addition, figure-specific mixed-effects models were performed to evaluate: (1) isolated SSE *versus* proximal emboli without SSE (Fig. 3); (2) embolus pattern among SSE-positive segments (Fig. 4); and (3) infarction status among SSE-positive segments (Fig. 5). For the comparison of embolus-pattern groups in Fig. 4, pairwise *post hoc* comparisons were performed using Tukey adjustment. Models were fitted using restricted maximum likelihood (REML) and implemented using the lme4, lmerTest, and emmeans packages in R. Associations between imaging parameters, including Mastora score, number of early-stage and manifest PI, number of SSE, and pIC, were assessed using Spearman’s rank correlation coefficient. A two-sided *p*-value < 0.05 was considered statistically significant.

## Results

### Study population

**Fig. 6:**
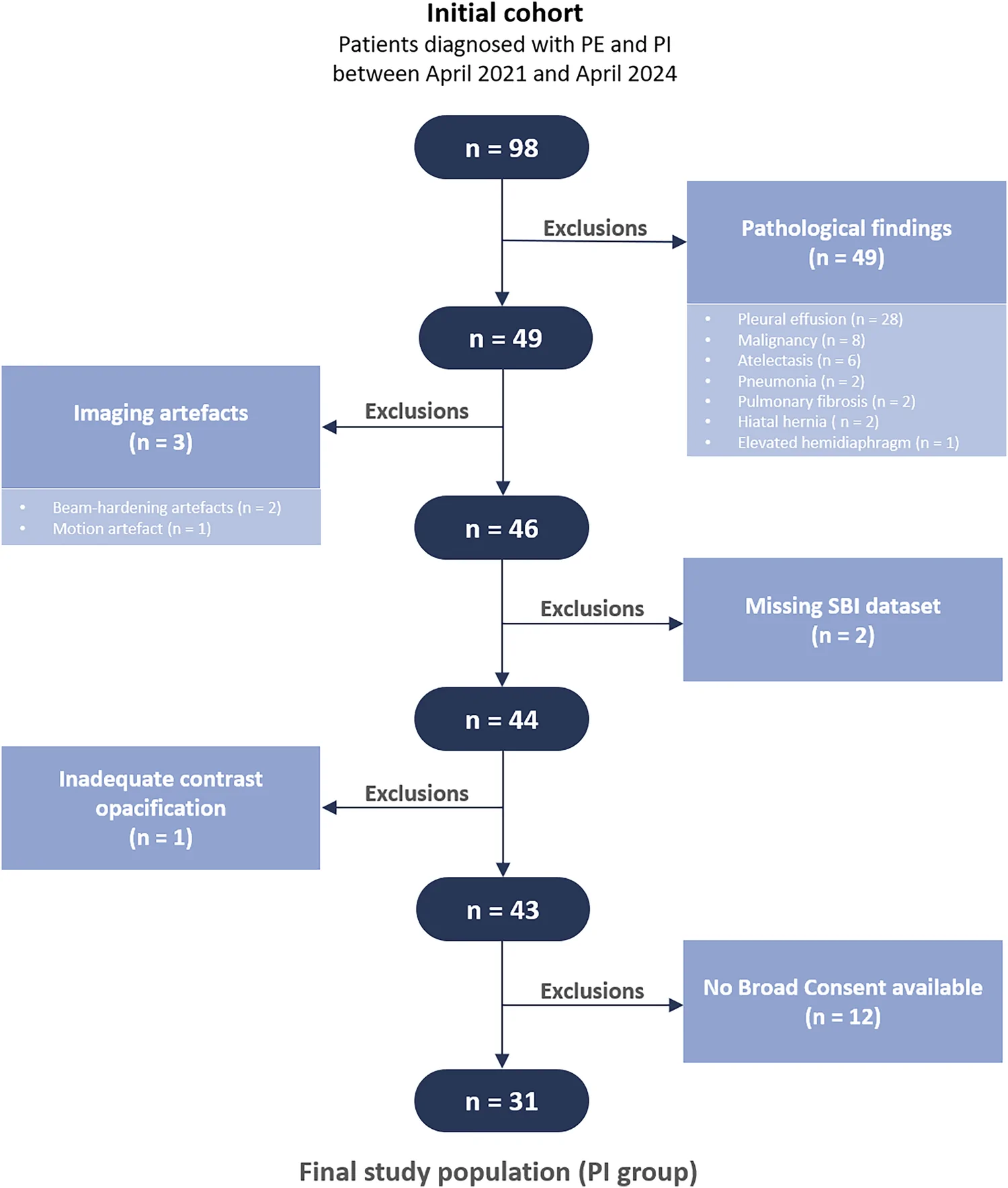
Flowchart of study population selection. Potential cases with PI were identified through a keyword-based search of radiology reports in the institutional radiology information system among SCTPA examinations performed for suspected acute PE. All identified examinations were subsequently reviewed to confirm the imaging findings. Of 98 identified cases with PE and PI, 67 were excluded based on predefined criteria, leaving 31 patients for the PI cohort. The control cohort comprised 31 patients without PE or PI selected from the same institutional SCTPA source population. PE, Pulmonary embolism; PI, Pulmonary infarction; SCTPA, Spectral computed tomography pulmonary angiography

Between April 2021 and April 2024, a total of 98 patients with SCTPA-confirmed acute PE and PI were identified. After applying the predefined inclusion and exclusion criteria, 31 patients remained eligible for the final analysis (Fig. 6).

A total of 67 patients were excluded due to predefined criteria. Parenchymal or anatomical limitations accounted for the majority of exclusions, including pleural effusion (*n* = 28), malignancy (*n* = 8), atelectasis (*n* = 6), pneumonia (*n* = 2), pulmonary fibrosis (*n* = 2), hiatal hernia (*n* = 2), and elevated hemidiaphragm (*n* = 1). Technical limitations included beam-hardening artefacts (*n* = 2), motion artefact (*n* = 1), missing or incomplete spectral base image datasets (*n* = 2), and inadequate contrast opacification (*n* = 1). After application of these criteria, the availability of documented GDPR-compliant broad consent was verified, and 12 patients were excluded due to missing consent.

The final study population comprised 62 patients, including 31 patients in the control group without PE or PI and 31 patients in the PI group with SCTPA-confirmed PE and at least one PI. The mean age of the control group was 62 ± 18.8 years (15 female), whereas the mean age of the PI group was 61 ± 15.5 years (13 female).

### Embolus distribution and thrombotic burden

In the PI group, vessel-based analysis using the Mastora scoring identified a total of 440 embolic occlusions (mean 14.2 per patient). The highest number of emboli was observed in the basal segments of the lower lobe, particularly in right segments 9 and 10 (*n* = 23 each) and left segment 8 (*n* = 22), followed by the left segments 9 and 10 (*n* = 19 each).

Thrombotic burden, as quantified by the Mastora score [25], was highest at the segmental arterial level. The most pronounced occlusions were observed in the right segmental arteries 9 and 10 (Mastora sum scores of 67 and 77, respectively) and in the left segmental artery 8 (score 65). At the lobar level, the lower-lobe arteries on both sides exhibited substantial thrombotic burden (right: 63; left: 65), whereas central vessels, including the pulmonary trunk, were only minimally affected (score 10). The distribution of embolic occlusions and corresponding thrombotic burden across pulmonary arterial branches is summarised in Table 1.

**Table 1:** Distribution of embolic occlusions and corresponding thrombotic burden across pulmonary arterial branches in the PI group (*n* = 31)

Vessel segment	Number of emboli (*n*)	Mastora score sum
Pulmonary trunk	5	10
Right lung		
Pulmonary artery	7	12
Interlobar artery	12	28
Upper lobar artery	16	50
Middle lobar artery	14	41
Lower lobar artery	19	63
Segmental artery 1	12	31
Segmental artery 2	12	33
Segmental artery 3	15	44
Segmental artery 4	13	37
Segmental artery 5	10	24
Segmental artery 6	18	56
Segmental artery 7	13	31
Segmental artery 8	17	56
Segmental artery 9	23	67
Segmental artery 10	23	77
Left lung		
Pulmonary artery	10	22
Interlobar artery	16	37
Upper lobar artery	13	44
Lingular artery	16	59
Lower lobar artery	16	65
Segmental artery 1	12	34
Segmental artery 2	9	30
Segmental artery 3	17	58
Segmental artery 4	13	47
Segmental artery 5	13	43
Segmental artery 6	16	44
Segmental artery 7*	-	-
Segmental artery 8	22	65
Segmental artery 9	19	43
Segmental artery 10	19	54

* Segment 7 of the left lung was anatomically absent in all patients

### Subsegmental emboli and pulmonary infarction distribution

In addition to the Mastora-based vessel analysis, SSE were assessed separately. A total of 589 lung segments were analysed; left segment 7 was anatomically absent in all patients and therefore excluded. Among the analysed segments, 120 demonstrated SSE.

**Fig. 7:**
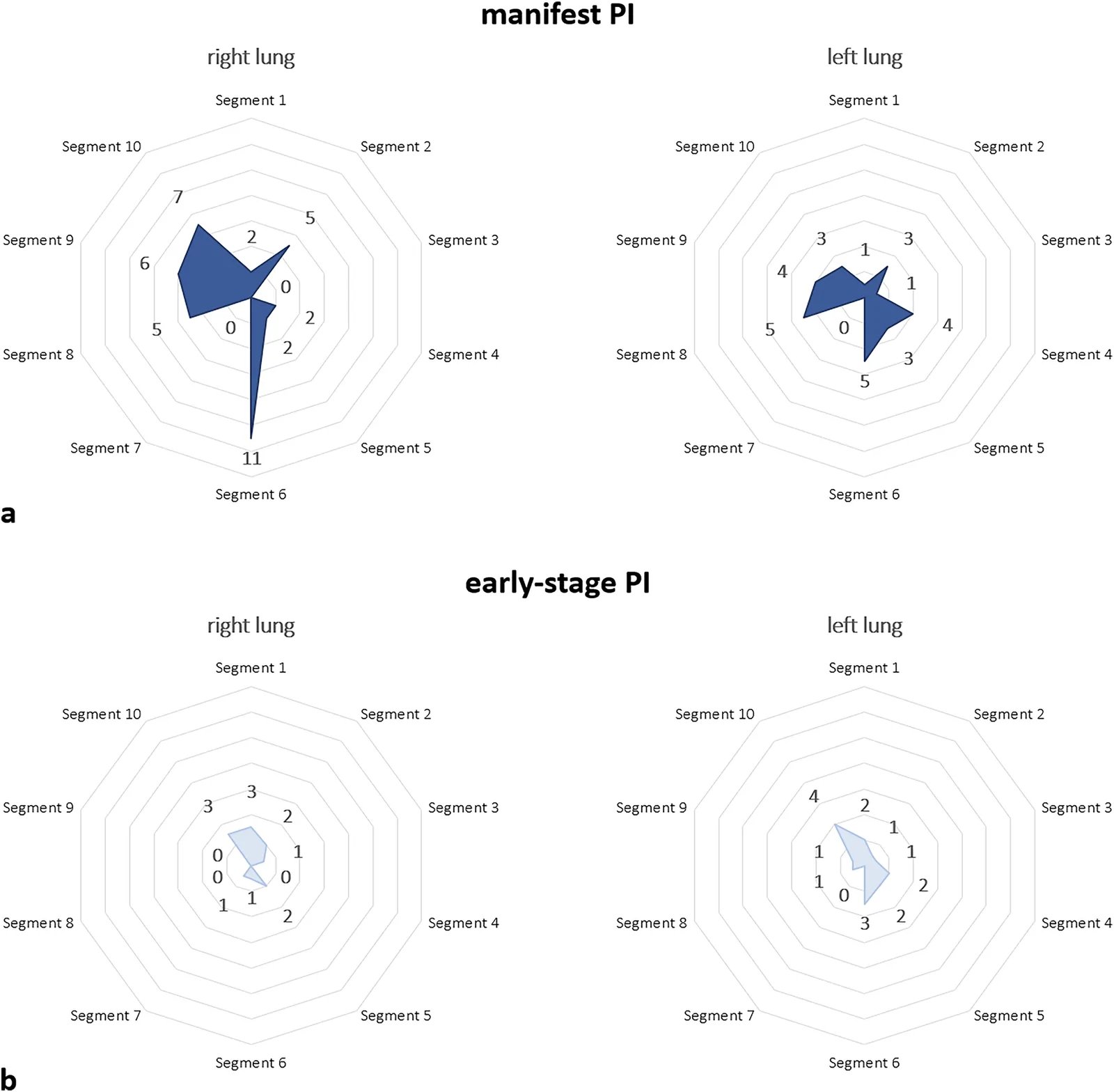
Segmental distribution of PI in the right and left lung. Radar charts illustrating the segmental distribution of PI by anatomical segment. **a** Manifest PI in the right and left lung. **b** Early-stage PI in the right and left lung. Values represent the absolute number of affected lung segments. PI, Pulmonary infarction

Of these 589 segments, 99 (16.8%) showed imaging evidence of PI, including 69 manifest and 30 early-stage infarctions. In this cohort, all PI occurred in segments affected by SSE, whereas no PI were observed in segments without SSE. Manifest PI occurred in 40 segments of the right lung and 29 segments of the left lung. In the right lung, the highest counts were observed in segment 6 (*n* = 11), followed by segments 10 (*n* = 7) and 9 (*n* = 6). In the left lung, segments 6 and 8 showed the highest counts (*n* = 5 each), followed by segments 4 and 9 (*n* = 4 each). Early-stage PI occurred in 17 segments of the left lung and 13 segments of the right lung. In the right lung, early-stage PI were most frequently observed in segments 1 and 10 (*n* = 3 each), whereas in the left lung they were most common in segment 10 (*n* = 4) and segment 6 (*n* = 3). The detailed segmental distribution of manifest and early-stage PI is shown in Fig. 7.

### Quantitative perfusion analysis and reliability

In the control group, segmental pIC values showed no significant left-right differences (F(1, 578) = 2.042; *p* = 0.154) but exhibited a pronounced cranio-caudal gradient, with significantly higher perfusion in basal compared with apical segments (right: F(9, 270) = 31.40; *p* < 0.001; *e.g*., segment 1 *versus* segment 10: *p* ≤ 0.001; left: F(8, 240) = 22.00; *p* < 0.001; *e.g*., segment 1 *versus* segment 10: *p* < 0.001). Measurement reproducibility was high, with intraclass correlation coefficient values of 0.870 in the control group and 0.905 in the PI group, indicating good to excellent agreement according to commonly used ICC interpretation guidelines [27].

### Perfusion analysis according to embolus characteristics and infarction status

To account for intra-patient clustering due to segment-based analysis, linear mixed-effects modelling was performed with patient ID included as a random intercept. In the global model, the presence of SSE was independently associated with reduced pIC ratio (β = -0.706, *p* < 0.001), and SE were also independently associated with lower pIC ratio (β = -0.137, *p* < 0.001), whereas LE and CE showed no significant independent associations (LE: β = 0.042, *p* = 0.263; CE: β = -0.038, *p* = 0.349). In a figure-specific mixed-effects model, segments with isolated SSE showed significantly lower pIC ratios than segments without SSE but with proximal emboli (SE, LE, or CE) (β = -0.577, *p* < 0.001; Fig. 3). Among SSE-positive segments, embolus pattern was significantly associated with pIC ratio overall (*p* = 0.010; Fig. 4). Tukey-adjusted pairwise comparisons showed that segments with isolated SSE had significantly higher pIC ratios than segments with additional proximal emboli, including SSE + SE (*p* = 0.017), SSE + SE + LE (*p* = 0.036), and SSE + SE + LE + CE (*p* = 0.020), whereas no significant differences were observed among the latter three groups. In contrast, among SSE-positive segments, infarction status was not significantly associated with pIC ratio (overall effect: *p* = 0.351; Fig. 5). Compared with infarct-free segments, neither early-stage PI nor manifest PI was associated with a significantly different pIC ratio in the mixed-effects model.

### Correlation analysis

**Fig. 8:**
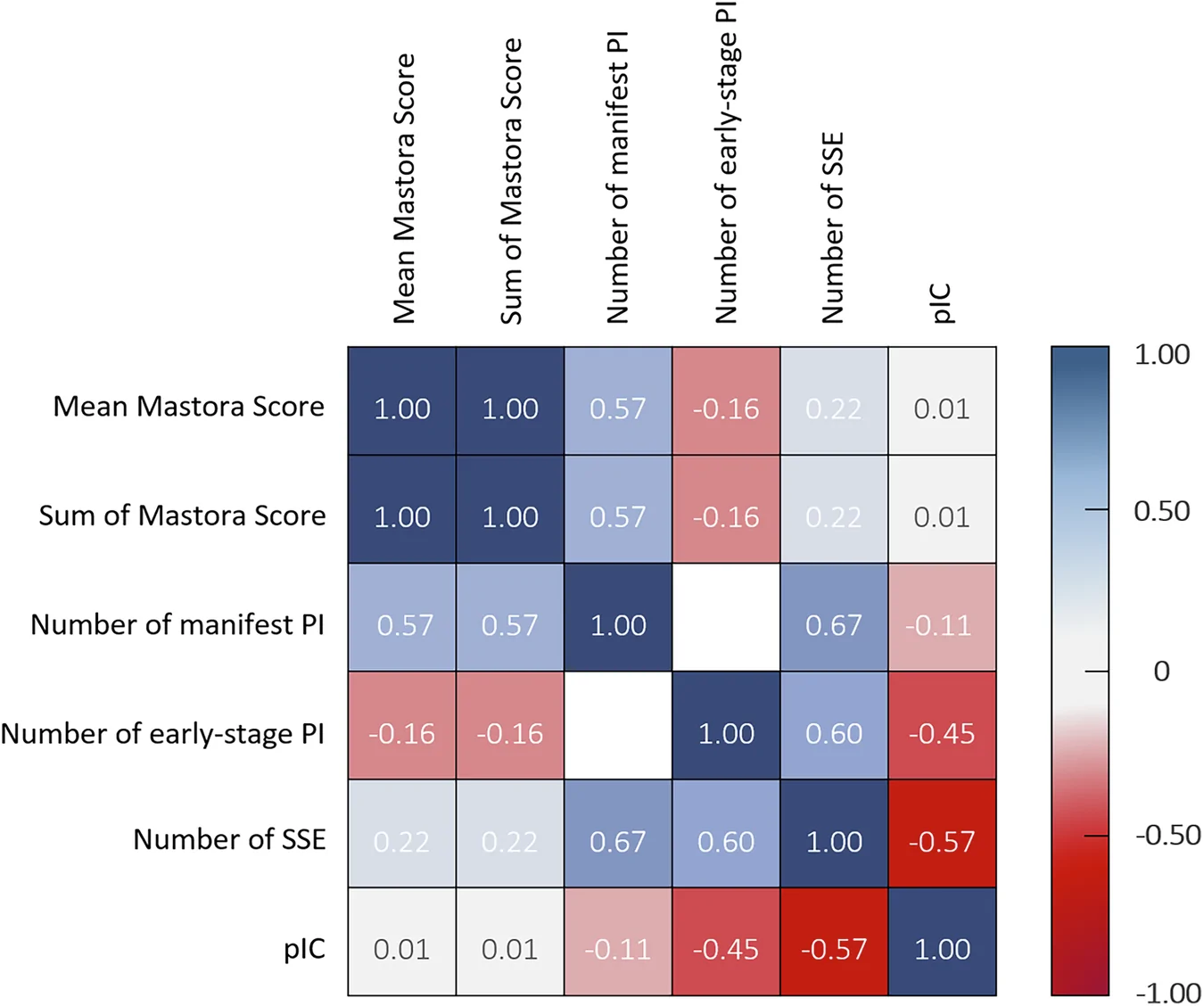
Spearman correlation matrix of imaging parameters. The figure shows the relationships between Mastora score, PI, SSE, and pIC using Spearman’s rank correlation. Positive and negative correlations are indicated by blue and red tones, respectively. PI, Pulmonary infarction; pIC, Peripheral iodine concentration; SSE, Subsegmental emboli

Spearman’s rank correlation analysis demonstrated a moderate-to-strong positive correlation between the number of SSE and the number of manifest PI (ρ = 0.665; *p* = 0.002) and a moderate negative correlation between SSE count and pIC ratio (ρ = -0.574; *p* = 0.010). Overall, thrombotic burden, as quantified by the summed Mastora score, showed a moderate correlation with the number of manifest PI (ρ = 0.566; *p* = 0.011) but was not associated with pIC ratio (ρ = 0.011; *p* = 0.963). The complete correlation matrix is presented in Fig. 8.

## Discussion

In this selected cohort, PI was observed exclusively in lung segments affected by SSE, whereas no PI was detected in segments without SSE. This consistent distribution pattern supports a close association between distally located vascular occlusions and the occurrence of PI. Mixed-effects modelling further demonstrated that SSE were independently associated with reduced pIC, even after accounting for intra-patient clustering. In addition, among SSE-positive segments, pIC did not differ significantly according to infarction status, indicating that relevant functional impairment of regional perfusion may already be present in the absence of morphologically evident infarction. Taken together, these findings suggest that regional hypoperfusion may be present even in the absence of morphologically visible structural parenchymal damage on CT.

These findings are consistent with the classical concept of the dual pulmonary blood supply of the lung. Virchow demonstrated in animal experiments that ligation of central pulmonary arteries alone does not result in PI, underscoring the compensatory capacity of the bronchial arterial circulation [28]. Hayek’s concept of so-called “shunt arteries”, which functionally connect the pulmonary and bronchial vascular systems, provides an anatomical basis for this compensatory mechanism. While perfusion deficits caused by central emboli can often be mitigated by bronchial arterial flow, terminal subsegmental arteries appear to possess only limited collateral capacity [17]. The present results support this concept and suggest that SSE may affect vascular territories in which compensatory mechanisms reach their functional limits, which may help explain their association with regional hypoperfusion and infarction.

Previous studies have reported a predominantly peripheral distribution of PI [6, 7, 13]. However, the specific contribution of SSE has not been fully characterised, presumably in part because of limited spatial resolution and the lack of quantitative functional perfusion assessment. The present study extends these observations by systematically linking SSE to regional hypoperfusion and infarction using spectral CT-derived quantitative perfusion metrics.

In the present study, pIC served as a functional marker of regional pulmonary perfusion deficits. Previous studies using SCTPA have demonstrated that iodine maps can reliably depict perfusion abnormalities [20–24] and may reveal such abnormalities before morphological changes become apparent [24]. To account for physiological differences in pulmonary perfusion between lung segments [29], perfusion values were normalised using segment-specific reference values derived from the control group. This approach allowed comparison of perfusion deficits relative to the expected regional baseline and reduced the influence of physiological segmental perfusion gradients. Nevertheless, residual interindividual differences in pulmonary perfusion may still have been present.

Against this methodological background, the present data indicate reduced pIC ratios in segments affected by SSE, regardless of whether the segment exhibits no PI, early-stage PI, or manifest PI. These findings support the interpretation of pIC as a marker of hypoperfusion rather than a marker of the infarction stage. This suggests that a relevant degree of perfusion impairment may occur early, whereas the development of structural parenchymal damage likely depends on temporal factors and additional modifying influences. In particular, variation in the interval between symptom onset and imaging may help explain why some segments with reduced pIC do not yet exhibit morphological signs of infarction. Consequently, while pIC enables the functional characterisation of perfusion-impaired lung regions, it cannot reliably predict which segments will ultimately progress to PI.

Mixed-effects modelling also provided a more differentiated view of embolus pattern and its relationship to peripheral perfusion. In the global model, SSE were independently associated with reduced pIC, and SE were also independently associated with reduced pIC, whereas LE and CE showed no significant independent associations. This suggests that impairment of subpleural perfusion is associated more with distal levels of vascular obstruction than with lobar or central embolic involvement.

Among SSE-positive segments, the embolus pattern was significantly associated with pIC ratio overall. Pairwise comparisons showed that segments with isolated SSE had higher pIC ratios than segments with additional proximal emboli, including SSE + SE, SSE + SE + LE, and SSE + SE + LE + CE, whereas no significant differences were observed among the latter three groups. These findings suggest that isolated SSE may represent a distinct subgroup with less pronounced peripheral perfusion impairment than SSE accompanied by additional proximal thrombotic burden. At the same time, the absence of significant differences among the three combined embolus-pattern groups argues against a simple stepwise decline in pIC with increasingly proximal extension once SSE are present. Rather, the data suggest that SSE may mark the point at which subpleural hypoperfusion becomes apparent, while additional proximal emboli may be associated with a greater perfusion deficit.

In contrast, when isolated SSE were compared with segments affected by more proximal emboli without subsegmental involvement, pIC ratios were significantly lower in the isolated SSE group. This finding further supports the concept that embolus location, rather than overall thrombotic burden alone, is an important factor associated with peripheral perfusion impairment. Distal arterial occlusions appear to affect vascular territories with limited collateral reserve, whereas more proximal obstruction may still permit residual pulmonary arterial flow and compensation *via* the bronchial circulation.

In line with this interpretation, the Mastora score showed only a moderate correlation with the number of manifest PI and was not associated with pIC. Although thrombotic burden reflects the overall extent of vascular obstruction, it appears to have limited ability to capture the functional relevance of emboli for the lung parenchyma, which may also depend on the anatomical level of vascular occlusion. A potential explanation is the relatively low degree of luminal obstruction in central pulmonary arteries, such as the pulmonary trunk and the main pulmonary arteries, in our cohort. In the Mastora score analysis, these vessels showed only limited thrombotic burden, suggesting that many proximal emboli were only partially occlusive. Partial obstruction may still allow sufficient residual pulmonary arterial flow and compensatory perfusion *via* the bronchial circulation.

Several limitations must be acknowledged.

First, potential cases with PI were identified through a keyword-based search of radiology reports. Although all identified examinations were subsequently reviewed to confirm the imaging findings, this selection strategy may have preferentially captured more typical, clearly visible, or explicitly reported cases of PI. Conversely, subtle, atypical, or unreported infarctions may have been missed. This may limit the generalisability of the findings and may have influenced the observed segment-level associations between SSE, pIC, and PI, despite the subsequent segment-based analysis.

Second, morphological image evaluation, including embolus localisation, assessment of the Mastora score, SSE detection, and classification of PI, was performed in consensus by the same readers and without formal blinding to embolus presence or to the study hypothesis. This design precluded assessment of interobserver variability for these morphological assessments. Moreover, the non-blinded, consensus-based interpretation may have introduced expectation bias, particularly because the main finding concerns the exclusive occurrence of PI in segments affected by SSE. Therefore, the strength of the observed SSE-PI association should be interpreted with caution and requires confirmation in future studies using independent, blinded readings and dedicated interobserver analyses.

Third, the retrospective, single-centre design and the relatively small sample size limit the generalisability of the findings. Fourth, PI and SSE diagnoses were based exclusively on CT morphology without histopathological confirmation. In the acute imaging setting, differentiation between alveolar haemorrhage and true PI remains challenging [4, 7]. Furthermore, no validated method exists to quantify the degree of occlusion in SSE, necessitating a dichotomous assessment.

In addition, the lack of temporal information regarding symptom onset and imaging limits conclusions regarding the dynamic evolution from hypoperfusion to infarction and precludes analyses based on time intervals between symptom onset and imaging. Moreover, the normalisation approach applied in this study does not fully account for interindividual variability in pulmonary perfusion and should be considered when interpreting the results. Clinical baseline characteristics, including relevant comorbidities, were not systematically available for all patients in this retrospective imaging-based cohort and could therefore not be analysed in a standardised manner. Although the analysis was performed at the segmental level, intra-patient clustering was addressed using mixed-effects modelling.

In summary, the findings of this study suggest that, in this selected cohort, SSE were closely associated with PI and regional perfusion impairment. SCTPA, through quantitative assessment of pIC, provides a functional complement to morphological embolus detection and enables characterisation of perfusion-impaired lung regions. However, pIC should be interpreted primarily as a marker of hypoperfusion rather than as a tool for predicting the infarction stage. Given the retrospective design, keyword-based case identification, and non-blinded consensus reading, the observed SSE-PI association should be interpreted with caution and requires confirmation in prospective studies with independent, blinded image assessment. Future studies should investigate whether regional perfusion deficits quantified by pIC correlate with established clinical risk markers in PE, such as the Pulmonary Embolism Severity Index (PESI) [30] or cardiac biomarkers, including troponin, and should clarify the potential role of pIC in clinical monitoring strategies.

## Data Availability

The datasets generated and/or analysed during the current study are not publicly available due to data protection regulation but are available from the corresponding author on reasonable request.

## Abbreviations

CE: Central emboli
CT: Computed tomography
CTPA: Computed tomography pulmonary angiography
GDPR: General Data Protection Regulation
LE: Lobar emboli
PE: Pulmonary embolism
PI: Pulmonary infarction
pIC: Peripheral iodine concentration
ROI: Region of interest
SCTPA: Spectral CT pulmonary angiography
SE: Segmental emboli
SSE: Subsegmental emboli
